# Current state of perinatal postmortem magnetic resonance imaging: European Society of Paediatric Radiology questionnaire-based survey and recommendations

**DOI:** 10.1007/s00247-020-04905-9

**Published:** 2020-12-23

**Authors:** Elspeth Whitby, Amaka C. Offiah, Susan C. Shelmerdine, Rick R. van Rijn, Michael Aertsen, Willemijn M. Klein, David Perry, Stacy K. Goergen, Christian Abel, Ajay Taranath, Dominic Gascho, Elka Miller, Owen J. Arthurs

**Affiliations:** 1 University of Sheffield and Sheffield Teaching Hospitals Foundation Trust, Jessop Wing, Tree Root Walk, Sheffield, S10 1SF UK; 2 Academic Unit of Child Health, University of Sheffield, Sheffield, UK; 3 Department of Radiology, Sheffield Children’s NHS Foundation Trust, University of Sheffield, Sheffield, UK; 4 Department of Clinical Radiology, Great Ormond Street Hospital for Children, London, UK; 5 UCL Great Ormond Street Institute of Child Health, London, UK; 6 Department of Radiology and Nuclear Medicine, Amsterdam UMC, University of Amsterdam, Amsterdam, The Netherlands; 7 Department of Radiology, University Hospitals KU Leuven, Leuven, Belgium; 8 Department of Radiology and Nuclear Medicine and Anatomy, Radboud University Medical Centre, Nijmegen, the Netherlands; 9 Radiology Department, National Women’s Health and Starship Children’s Hospital, Auckland City Hospital, Auckland, New Zealand; 10 Monash Imaging, Clayton, Victoria Australia; 11 School of Clinical Sciences, Monash University, Clayton, Victoria Australia; 12 Department of Medical Imaging, John Hunter Hospital, Newcastle, New South Wales Australia; 13 Department of Medical Imaging, Women’s and Children’s Hospital, North Adelaide, South Australia Australia; 14 University of Adelaide, Adelaide, South Australia Australia; 15 Zurich Institute of Forensic Medicine, University of Zurich, Zurich, Switzerland; 16 Department of Medical Imaging, Children’s Hospital of Eastern Ontario, University of Ottawa, Ottawa, Canada

**Keywords:** Consensus recommendations, Foetal, Imaging, Magnetic resonance imaging, Perinatal, Postmortem, Protocol, Survey

## Abstract

**Background:**

Postmortem magnetic resonance imaging (MRI) in perinatal and childhood deaths is increasingly used as a noninvasive adjunct or alternative to autopsy. Imaging protocols vary between centres and consensus guidelines do not exist.

**Objective:**

Our aim was to develop practical, standardised recommendations for perinatal postmortem MRI.

**Materials and methods:**

Recommendations were based on the results of two surveys regarding local postmortem MRI practices sent electronically to all 14 members of the European Society of Paediatric Radiology (ESPR) Postmortem Imaging Task Force and 17 members of the International Society of Forensic Radiology and Imaging Task Force (25 different centres).

**Results:**

Overall, 11/14 (78.6%) respondents from different institutions perform postmortem MRI. All of these centres perform postmortem MRI for perinatal and neonatal deaths, but only 6/11 (54.5%) perform imaging in older children.

**Conclusion:**

We propose a clinical standard for postmortem MRI sequences plus optional sequences for neuroimaging and cardiac anatomy depending on available scanning time and referral indications.

## Introduction

Paediatric postmortem magnetic resonance imaging (MRI) was first reported in 1996 by Brookes et al. [1]. Since then, there have been numerous studies by research groups worldwide [2–4] assessing the value of paediatric postmortem MRI in addition to or instead of autopsy. Initially, the central nervous system was assessed by paediatric postmortem MRI with high diagnostic accuracy [5]; more recently, whole-body imaging including paediatric postmortem MRI has been used to predict organ weights [6] and guide biopsy [7, 8]. Researchers have also investigated higher field MRI compared to conventional MRI [9] to evaluate small structures such as the heart [10]. The largest study to date of 400 cases showed high diagnostic accuracy in foetuses and stillbirths [11], particularly for the brain, abdomen and heart [12–14].

While several centres now use paediatric postmortem MRI in routine clinical practice, there are no consensus guidelines for paediatric/perinatal postmortem MRI protocols. The European Society of Paediatric Radiology (ESPR) set up a task force in 2016 to establish the extent and nature of current clinical utilisation and to develop minimum standards for clinical radiology practice. In this manuscript, we outline a questionnaire-based survey of postmortem MRI protocols used by ESPR and International Society of Forensic Radiology and Imaging (ISFRI) members, and generate a consensus view on a clinical standard protocol.

## Materials and methods

This was a prospective international, multicentre survey of current paediatric postmortem MRI practices and imaging protocols. Institutional ethical review board approval was not required as no patient data were shared or accessed.

An initial fact-finding email (Appendix) was sent to all 17 members of the ISFRI and 14 members of the ESPR Postmortem Imaging Task Force in January 2018 consisting of 5 questions related to paediatric and perinatal postmortem MRI, age ranges and sequences used. Members who did not perform foetal, neonatal or paediatric postmortem MRI were excluded from further correspondence. Two short follow-up surveys were subsequently distributed by email. The lead author (E.W.) developed and designed the two subsequent surveys based in part on the responses to the initial questions.

An initial, 13-question free-text survey (Appendix) was sent to members in March 2018 asking for information regarding the type of MR scanner used, sequence choice, typical duration of scanning and selection of cases.

A second, 10-question multiple-choice survey (Appendix) was sent in January 2019 regarding members’ paediatric postmortem MRI experience, training and the impact of their paediatric postmortem MRI service on autopsy referrals. All responses received by March 2019 were included for descriptive analysis.

Survey responses were presented at the Postmortem Imaging Task Force session of the annual ESPR conference in June 2018 in Berlin, Germany [15], and an update was presented at the annual ESPR conference in May 2019 in Helsinki, Finland [16].

Based on the survey results, a recommended clinical standard paediatric postmortem MRI protocol was proposed at the Helsinki meeting and further developed by the task force, with additional imaging considerations adapted from the latest published research on paediatric postmortem MRI.

The final version of this manuscript was circulated among authors in 2020 for consensus approval and formally endorsed by the ESPR board before submission for publication.

## Results

In total, 25 international imaging centres were invited to complete the survey questionnaires. Fourteen responses (56%) were received. In 2/14 (14%) centres, paediatric/perinatal postmortem MRI was not performed and 1 of the 14 (7%) centres did not fully complete the questionnaire. Therefore, completed surveys from 11/14 centres (78%) were included in our final analysis. Of the completed questionnaires, 13 (93%) were completed by radiologists who conducted and reported postmortem magnetic resonance imaging examinations and 1 (7%) was completed by a technologist.

The responses originated from the following countries and continents, including:

- Europe: 6/11 (54%); United Kingdom (2, 18%), The Netherlands (2, 18%), Belgium (1, 9%) and Switzerland (1, 9%).
- Oceania: 4/11 (36%); Australia (3, 27%), New Zealand (1, 9%).
- North America: 1/11 (9%); Canada (1, 9%).

### Referrals and reporting (Table 1)

All centres performed paediatric postmortem MRI on a scanner located in their main radiology department. None of the centres had a dedicated MRI scanner solely for postmortem imaging, nor one located in the mortuary or pathology department.

**Table 1:** Summary responses received from all 11 respondents to the survey

**Country**	**Years performing PMMR**	**Reporting** **training**	**Time taken to scan**	**PMMR cases per year**	**Funding**	**Scanner** **location**	**Reporter**	**Case referral**	
								**Perinatal (foetal up to birth) and neonatal cases (28 days or less)**	**Paediatric cases (1 year to 18 years)**
**Oceania**									
Australia	5	No	NS	NS	None	Radiology	Radiologists	Yes	No
Australia	11	No	NS	14	Paid by referrer	Radiology	Radiologists	Yes	No
Australia	9	No	30–40 min	90–100	None	Radiology	Radiologists	Yes	No
New Zealand	14	No	NS	20	Case-by-case basis	Radiology	Radiologists	Yes	No
**North America**									
Canada	11	No	40 min	20	None	Radiology	Radiologists	Yes	No
**Europe**									
Belgium	6	No	90–120 min	>50	None	Radiology	Radiologists	Yes	No
The Netherlands	12	No	NS	5–10	None	Radiology	Radiologists	Yes	Yes
The Netherlands	10	Comparison study	30–60 min	50–60	None	Radiology	Radiologists	Yes	Yes
Switzerland	9	Zurich virtopsy course	60–90 min	20	State attorney (forensic); research grant	Forensic medicine and imaging	Radiologists or forensic pathologist	Yes	Yes
United Kingdom	20	Research study	20–40 min	50	Postmortem funding	Radiology	Radiologists	Yes	No
United Kingdom	17	In house	90 min	100–120	Autopsy payment	Radiology	Radiologists	Yes	Yes

*NS* not stated, *PMMR* postmortem magnetic resonance imaging

Referrers varied between centres but usually included specialists in obstetrics and gynaecology or pathology. Infrequently, a radiologist or parents requested paediatric postmortem MRI. In all centres, the referring doctor communicated the results of the paediatric postmortem MRI to the parents.

Radiologists were responsible for reporting in all centres, with a forensic pathologist involved for forensic cases at only one centre. Despite this, none of the radiologists had formal training in paediatric postmortem MRI. One (1/11, 9.1%) radiologist had received in-house training from a senior colleague, whilst 2/11 (18.2%) centres based their training on research studies undertaken before their clinical services were implemented.

The median duration of paediatric postmortem MRI service provision across all centres was 11 years (range: 5–20 years). The number of cases performed annually ranged from 10 to 120 (mean: 40, median: 20). Most centres believed demand was stable, whereas 4/11 (36.4%) centres believed demand was increasing.

All centres provided a paediatric postmortem MRI service for terminations of pregnancy, stillbirths and neonatal deaths. Only 6/11 (54.5%) centres offered paediatric postmortem MRI services for older children (>1 year). In the Netherlands, this is for the unexpected deaths of children in infancy. Although there is widespread opinion that paediatric postmortem MRI may reduce invasive autopsies, and 4/11 (36.4%) respondents believed overall conventional autopsy rates in their region were decreasing, none of the respondents had evidence to support this.

None of the centres provide a fully funded, routine service for paediatric postmortem MRI, with the majority performed on a case-by-case basis and the resulting costs absorbed by global hospital or department budgets. Two centres based in the United Kingdom receive some funding as part of the United Kingdom postmortem funding stream (i.e. a portion of the government fee-for-service payment for all paediatric or perinatal conventional autopsies). In the Netherlands, there is government funding for unexpected/unexplained deaths of children from infancy to their 18th birthday.

### Imaging protocols (Table 2)

In only 1/11 (9.1%) centres, located in Belgium, did the radiologist personally acquire their own MR images. In the other 10/11 (90.9%) centres, a radiographer or technologist performed this role. Fourteen scanners were used: 3 (27.2%) centres used both 3-tesla (T) and 1.5-T scanners, 3 (27.2%) centres used only a 1.5-T scanner and 5 (45.4%) centres used only a 3-T scanner. One centre also had access to a 7-T research scanner.

**Table 2:** Consensus clinical standard sequences for postmortem foetal brain and body examinations

**Sequence**	**FOV (mm)**	**Slice thickness (mm)**	**Gap**	**TR (ms)**	**TE (ms)**	**Averages (NEX/NSA)**	**Voxel size (mm)**	**Duration of sequence (min)**
**T2 sequences (brain)**								
2-D T2 TSE 3 planes	180×100	3	0.3	3,000	150	2	0.8×0.6×3.0	1.56
SWI (if haemorrhage suspected)	180×148	4	0	31	7.2	4	0.6×0.6×4.0	1.32
**T2 sequences (body)**								
T2 TSE 3 planes	150×119	3	0	3,870	120	2	0.55×0.59×3.0	3.13
3-D T2 TSE	200×200	0.8	0	3,500	275	2	0.8×0.8×0.8	6.2
**T1 volumetric sequence (brain and body)**								
T1 3-D	120×100	2.0	0	7	2.46	1	1.3×1.3×2.0	1.09

Two-dimensional (2-D) turbo spin echo (TSE) can be used for body images in three planes or a volume sequence

*3-D* three-dimensional, *FOV* field of view, *NEX* number of excitations, *NS* not stated, *NSA* number of signal averages, *SWI* susceptibility-weighted imaging, *TE* echo time, *TR* repetition time

All centres performed T2-weighted sequences in three orthogonal planes, with the addition of at least one T1-weighted sequence, usually in the axial plane for brain and body imaging. Other additional sequences were performed by some institutions, the most frequent being a susceptibility-weighted imaging (SWI) sequence or T2* for detecting haemorrhage (4/11, 36.4%). All radiologists said T2-weighted images were the most useful for interpretation as they provided the most anatomical detail, while diffusion-weighted imaging (DWI) was deemed the least useful.

The average time for a complete paediatric postmortem MRI examination varied between 20 and 120 min (mean: 40 min), depending on patient and sequences employed.

The following were among the survey responses:

1. The importance of discussing the findings with the pathologist, if an autopsy was to take place, to ensure that any abnormality seen on imaging was included in histological sections.
2. The importance of taking care when reporting as often there are unexpected findings.
3. The importance of performing postmortem MRI within 24 h of the request to ensure the parents receive information as quickly as possible.
4. The value of allowing technicians/radiographers to choose the timing to fit around their needs, as this reduces the stress of the situation.
5. The value of an international program to help support countries and demonstrate the value of paediatric postmortem MRI to governments and insurance companies.
6. One centre has shown that paediatric postmortem MRI is not as accurate as intrauterine foetal MRI for central nervous system cases [17], a finding supported by another study [18]. Consequently, the centre is now using intrauterine foetal MRI as a first step in postmortem investigations when there is an intention to terminate the pregnancy due to a foetal cranial abnormality.

## Discussion

This prospective survey of a small number of centres performing paediatric postmortem MRI has revealed the common aspects of and variations in referral, technical and reporting practices of paediatric/perinatal postmortem MRI practices internationally and a consensus basic clinical scanning protocol has been developed by the survey participants and task force members. These recommendations will allow for future standardisation of image acquisition and improved ease of multisite reporting.

The recommended imaging sequences are easily adaptable to different patient sizes and scanner manufacturers. Members of the ESPR task force are willing to support other centres in developing paediatric postmortem MRI services and to review imaging scans if requested.

In addition to the standard clinical protocol, specific sequences for brain imaging should include blood-sensitive sequences (e.g., T2* weighted or SWI). Other sequences such as fluid-attenuated inversion recovery (FLAIR) may be included, although their additional diagnostic value in paediatric postmortem MRI is unclear. For body imaging, a three-dimensional (3-D) T2-weighted constructive interference steady state (CISS) sequence provides high-resolution cardiac anatomical imaging, although it requires an additional 30 min of scanning time and may be best reserved for cases where there is a suspected cardiac anomaly [19]. Whole-body short tau inversion recovery (STIR) imaging of the brain may also be helpful, particularly as many radiologists are familiar with this sequence in whole-body live paediatric imaging and believe it provides excellent visualisation of the lamination pattern in the foetal brain. Table 3 details these additional sequences.

**Table 3:** Additional (optional) sequences for the postmortem MRI examination

**Sequence**	**FOV (mm)**	**Matrix**	**Slice thickness (mm)**	**Gap**	**TR (ms)**	**TE (ms)**	**Averages (NEX/NSA)**	**Voxel size (mm)**	**Duration (min)**
**Additional ‘optional’ brain sequences**									
SWI	180×148	300×247	4	0	31	7.2	4	0.6×0.6×4.0	1.32
T2*	150×122	168×134	3	0	Shortest	23	2	0.9×0.9×3.0	3.56
FLAIR (long TR)	150×117	220×136	3	0	11,000	140	3	0.75×0.85×3.0	4.46
STIR	200×200	216×320	4	0	6,180	14 and 115	1	06.×0.7×4.0	3.19
**Additional ‘optional’ body sequences**									
STIR	200×200	216×320	4	0	6,180	14 and 115	1	0.6×0.7×4.0	3.19
3-D CISS, T2 (for cardiac pathologies, cover heart and entire lungs)	150×150	192×256	0.6	0	5.6	2.5	10	0.6×0.6×0.6	29.26

*3-D* three-dimensional, *CISS* constructive interference in a steady state, *DWI* diffusion-weighted imaging, *FLAIR* fluid-attenuated inversion recovery, *FOV* field of view, *NEX* number of excitations, *NSA* number of signal averages, *STIR* short tau inversion recovery, *SWI* susceptibility-weighted imaging, *TE* echo time, *TR* repetition time

The types of coils used were not included in our survey questions, but most centres select the smallest coil the body will fit to maximize signal and obtain as much coverage of the body as possible. Further work is underway to assess the best methods to prepare a body for postmortem imaging, and which imaging modality to use and when in the foetal, neonatal and paediatric age groups.

In all centres, the paediatric postmortem MRI reporting is performed by radiologists, with the majority having no formal training in postmortem imaging. Skills were either self-taught by adapting them from live paediatric or intrauterine foetal imaging experiences or via a postmortem imaging research environment before starting clinical services. There is, therefore, a need for dedicated training programs and guidelines, particularly if more centres are to be encouraged to provide paediatric postmortem imaging services.

The only published guidelines that we are aware of are the Dutch guidelines for clinical foetal–neonatal and paediatric postmortem radiology [20]. These aim to guide the choice of imaging modality for the size of the body and also take into account the clinical details at presentation.

It is important to note that whilst we included task force members of ESPR and ISFRI, other radiologists, pathologists and medical professionals may perform paediatric postmortem imaging outside these organisations and, therefore, may have been excluded. Nevertheless, this document provides a starting point for consensus guidelines from the current field of expertise.

## Conclusion: consensus recommendation

All centres supported the notion of provision of a consensus clinical standard set of paediatric postmortem MRI sequences that should be included in every examination. This was defined as a basic clinical protocol that:

- Would provide good quality images across all age ranges,
- Is readily achievable in the majority of centres, with minor modifications using any MR scanner model, and
- Allows for multiplanar analysis of key anatomy.

Based on current practices collated from survey responses, the basic clinical standard protocol developed through group consensus included:

- T2-weighted images in three orthogonal planes (axial, coronal, sagittal)
- 3-D T1-weighted isovolumetric sequence
- SWI or T2*-weighted imaging in cases with possible haemorrhage.

The imaging parameters could be adapted to cover the whole body in a single acquisition (brain and torso) or divided into separate brain and torso (thorax and abdomen) acquisitions, largely determined by patient size (Table 2), achievable in under 30 min.

We hope that this will standardise and improve clinical practice, reporting, collaboration and second opinion work, both in clinical and medicolegal practice settings.

## Appendix

### Initial fact-finding email

- Do you currently perform PMMR?
- What age group do you perform PMMR for?
- What sequences do you use?
- How long does your protocol take?
- How many do you perform a month?

Any other information?

### Questions in second follow-up email

- How long have you been performing PMMR for?
- Are you doing PMMR for the brain or whole body or both?
- How long in total does your PMMR protocol take?
- Why have you chosen the sequences you use?
- Which sequences do you find most useful?
- Are there any sequences you do not find useful?
- Do you do 3D volume imaging? Is this to save time? Do you re format in all 3 planes?
- Do you image all cases referred or selected cases? Why?
- Is the number of cases you receive increasing overtime?
- Are you restricted by any local rules?
- Are you restricted by the cost of imaging?
- Are there any aspects you feel are really important to your practice, e.g. certain sequences, reformats, turnaround time, communication of results?
- Do you have anything in your service that you feel is really good practice and would like to see other people use?

### ESPR Postmortem Task Force — detailed questionnaire

1. Do you perform postmortem MRI?

- How long have you been performing PMMR?
- Did you have any training?
- How many do you do an average per year?
- Is this increasing?
- How is this funded in your country?

2. What is the reason you perform PMMR?

- Is it at parents’ request?
- Does the state suggest it?
- Does the coroner or equivalent accept it?

3. Do you know if this has had an impact on the number of full autopsies performed?

- Has this increased or decreased the number of cases that have investigations performed after death?
- Do you still perform autopsy or limited autopsy in cases that have had PMMR?
- What is the overall percentage of cases that have post death investigations in your centre and is this the same, more or less than in your country?

4. Would it be helpful to standardise the sequences used?

- Local standards
- National standards
- International standards
- Have a minimum standard?

5. What is your scanner manufacturer?
6. Where is your scanner located, e.g. radiology department, mortuary, off site?
7. What age group of cases do you scan? Please tick all that apply:

- Foetal
- Terminations of pregnancy (TOP)
- Stillbirths
- Neonates
- All paediatric cases

Any additional details?

8. Who reports your images?

- Radiologist
- Radiographer
- Pathologist

9. Who obtains the images?

- Technicians
- Radiographers
- Radiologists

10. How do the parents obtain the results?
